# Mechanistic Insight into the role of Vitamin D and Zinc in Modulating Immunity Against COVID-19: A View from an Immunological Standpoint

**DOI:** 10.1007/s12011-023-03620-4

**Published:** 2023-03-09

**Authors:** Nuzhat Ahsan, Mohammad Imran, Yousuf Mohammed, Fatme Al Anouti, Mohammad Idreesh Khan, Tanushree Banerjee, Mohd Adnan, Fauzia Ashfaq, Marek Kieliszek, Syed Amir Ashraf, Afrozul Haq

**Affiliations:** 1 Quantum Biphotonics Division, Quantlase Laboratory LLC, Abu Dhabi, UAE; 2 Therapeutic Research Group, Frazer Institute, Faculty of Medicine, The University of Queensland, Brisbane, 4102 Australia; 3 College of Natural and Health Sciences, Zayed University, Abu Dhabi, UAE; 4 Department of Clinical Nutrition, College of Applied Health Sciences in Ar Rass, Qassim University, Ar Rass, 51921 Saudi Arabia; 5 Infosys Ltd. SEZ Unit VI, Plot No. 1, Rajiv Gandhi Infotech Park, Hinjawadi Phase I, Pune, Maharashtra 57 India; 6 Department of Biology, College of Science, University of Haʼil, P.O. Box 2440, Haʼil, Saudi Arabia; 7 Department of Clinical Nutrition, College of Applied Medical Sciences, Jazan University, Jazan, 45142 Saudi Arabia; 8 Department of Food Biotechnology and Microbiology, Institute of Food Sciences, Warsaw University of Life Sciences—SGGW, Nowoursynowska 159 C, 02-776 Warsaw, Poland; 9 Department of Clinical Nutrition, College of Applied Medical Sciences, University of Haʼil, Haʼil, Saudi Arabia; 10 H.A. University, Imphal, Manipur India

**Keywords:** COVID-19, SARS-CoV-2, Vitamin D, Innate immunity, Adaptive immunity, Zinc

## Abstract

The pathophysiology of coronavirus disease-19 (COVID-19) is characterized by worsened inflammation because of weakened immunity, causing the infiltration of immune cells, followed by necrosis. Consequently, these pathophysiological changes may lead to a life-threatening decline in perfusion due to hyperplasia of the lungs, instigating severe pneumonia, and causing fatalities. Additionally, severe acute respiratory syndrome coronavirus 2 (SARS-CoV-2) infection can cause mortality due to viral septic shock, resulting from unrestrained and backfiring immune reactions to the pathogen. Sepsis can cause premature organ failure in COVID-19 patients, as well. Notably, vitamin D and its derivatives and minerals, such as zinc and magnesium, have been reported to improve the immune system against respiratory illnesses. This comprehensive review aims to provide updated mechanistic details of vitamin D and zinc as immunomodulators. Additionally, this review also focuses on their role in respiratory illnesses, while specifically delineating the plausibility of employing them as a preventive and therapeutic agent against current and future pandemics from an immunological perspective. Furthermore, this comprehensive review will attract the attention of health professionals, nutritionists, pharmaceuticals, and scientific communities, as it encourages the use of such micronutrients for therapeutic purposes, as well as promoting their health benefits for a healthy lifestyle and wellbeing.

## Introduction

Severe acute respiratory syndrome coronavirus 2 (SARS-CoV-2), the novel coronavirus behind the pandemic affecting millions of lives and livelihoods around the world, is a single positive-stranded ribonucleic acid (RNA) virus, which belongs to the family Coronaviridae. It primarily causes respiratory tract infections. In addition, it may be complicated by liver, brain, and gastrointestinal tract diseases [1, 2]. The severity and lethality of the disease have been reported to be due to an intensified inflammation marked by the infiltration of immune cells, necrosis, and edema of lung tissue, causing impaired pulmonary oxygen exchange. COVID-19 manifests predominantly as pneumonia, where an aggressive immune reaction can lead to a “cytokine storm” primarily in the lungs. This immune response involves several cytokines, tumor necrosis factor alpha (TNF-α), interleukin-1 beta (IL-1β), interleukin-8 (IL-8), interleukin-12 (IL-12), and interleukin-10 (IL-10), monocyte chemoattractant protein 1 (MCP1) and macrophage inflammatory protein 1A (MIP1A), with interleukin-6 (IL-6) leading the pathological cascade. The inflammatory response leads to what is now termed microvascular COVID-19 lung vessel obstructive thrombo-inflammatory syndrome (microclots) [3, 4]. Additionally, in older patients, a change in the plasma levels of lymphocytes, thrombocytes, C-reactive protein, and lactate dehydrogenase enzyme can lead to sepsis, aggravation of the symptoms, and a prolonged need for medical care [5, 6]. Clinically, COVID-19 shows a broad range of presentations, ranging from being asymptomatic and non-septic to mild upper respiratory tract infection (URTI), pneumonia, and acute respiratory distress syndrome (ARDS), sepsis, and death. It should be noted that when the clinical frailty scores (CFS) are calculated to assess the risk severity, factors such as age, comorbidities, and sepsis should be attuned [7].

As we face an absence of specific treatment, while the disease continues to spread, re-emerge, and become a vicious circle of infections and reinfections, our understanding of the mechanisms reinforces the fact that a tolerable immune response is fundamental to preventing and treating this viral infection. Therefore, it has become very important for the scientific community to explore existing pharmacological agents that strengthen the immune activity. Vitamins and minerals have been reported to aid the proper functioning of the immune system and protect the host immune response. Therefore, owing to the significant properties of vitamins and minerals as immunomodulators, this review presents an update on the role of vitamin D and zinc as immunomodulators and suggests mechanistic approaches to address the COVID-19 pandemic.

**Fig. 1:**
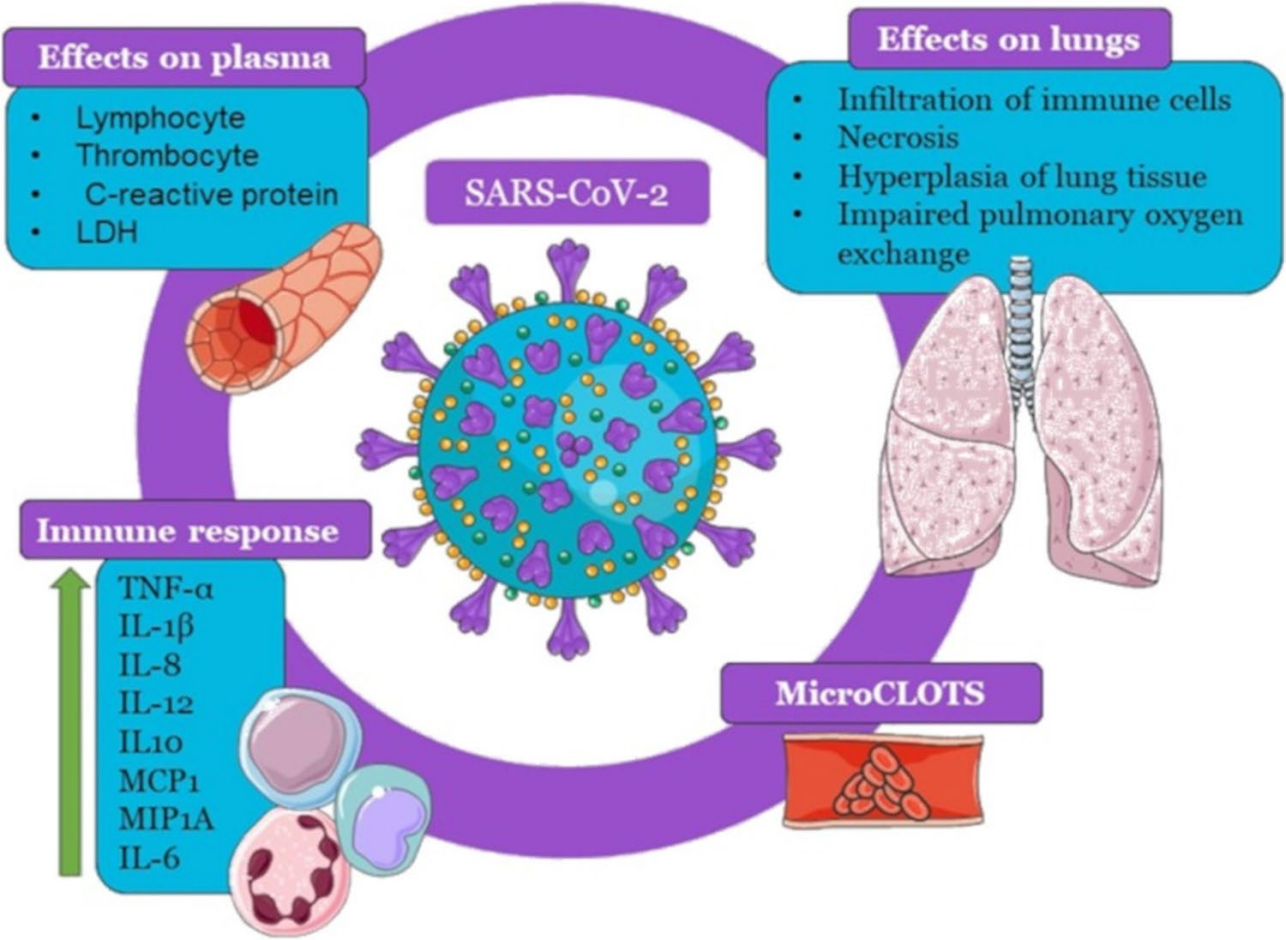
Effects of Sars-CoV-2 in the human body

**Fig. 2:**
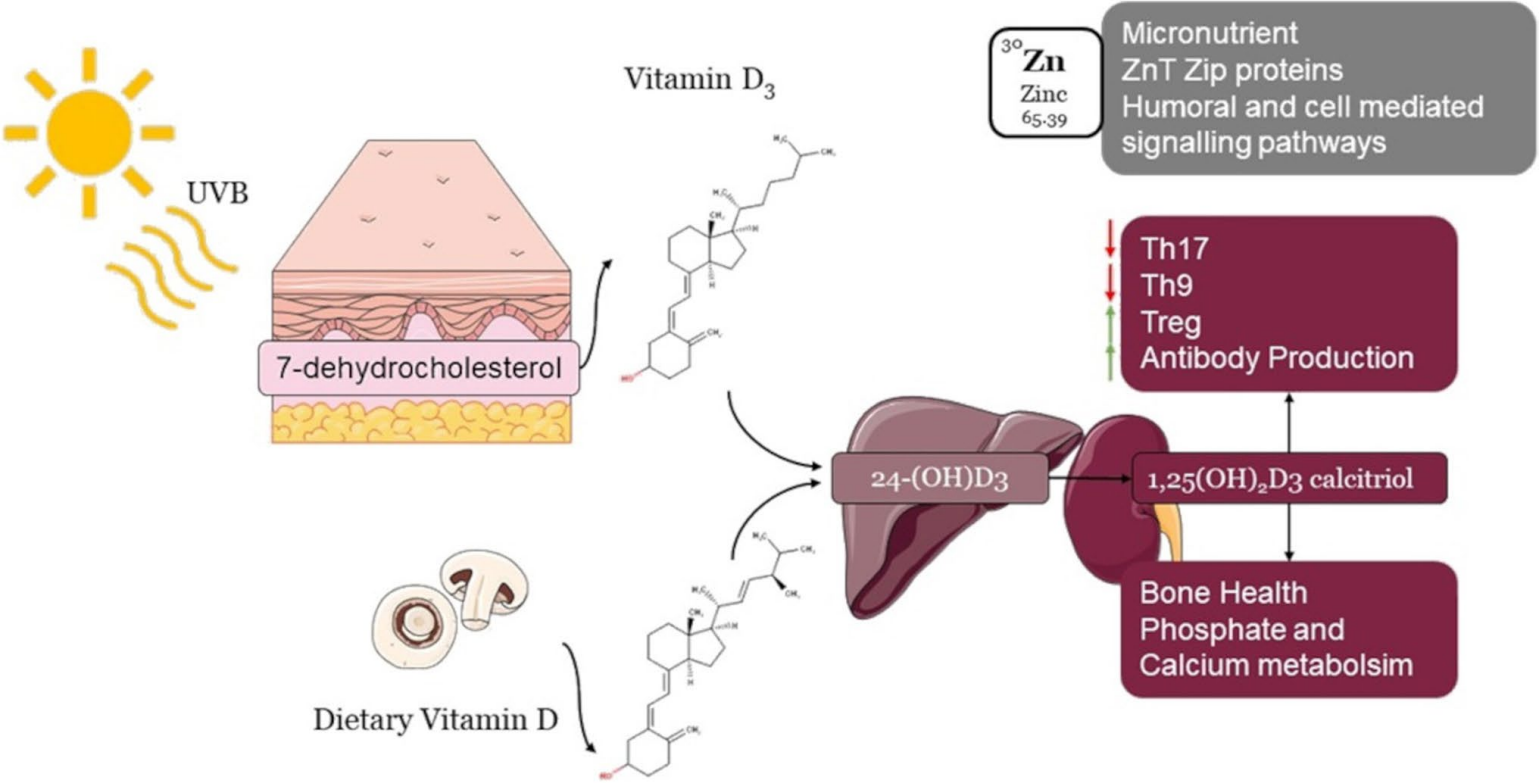
Synthesis and effects of vitamin D and zinc

Vitamin D was discovered in 1920, followed by the discovery of the vitamin D receptor (VDR) in 1969. At first, vitamin D was known to be a nutritionally essential steroid vitamin; later, it was recognized as an endocrine hormone and was further explored for its as immunomodulatory properties [8]. In humans, vitamin D is present in two physiological forms, vitamin D2 (ergocalciferol) and vitamin D3 (cholecalciferol). Vitamin D3 is synthesized in the skin through the reaction of 7-dehydrocholesterol by ultraviolet B radiation (UVB) to form provitamin D3. Moreover, vitamin D2 is photochemically synthesized in plants, and among the plant sources, mushrooms exposed to radiation have been reported to have a high yield of vitamin D2 [9]. Furthermore, being a nutritionally essential steroid vitamin, vitamin D has been ranked as one of the important immunomodulators, along with its pleiotropic biological significance. Vitamin D supplementation can decrease the risk factors for major diseases, i.e., nutritional rickets, cancer, type 2 diabetes mellitus, and cardiovascular diseases Fig. 1. Moreover, it also improves lung functioning, delays bone loss, and prevents bone fractures [8]. The liver contains the 25-hydroxylase enzyme, which metabolizes vitamin D3 to 25(OH)D3, the circulating form and the serum marker [10–12]. 25(OH)D3 is acted upon by a kidney mitochondrial enzyme CYP27B1 (25-hydroxyvitamin D3 1-α-hydroxylase) to form biologically active 1,25(OH)_2_D3 calcitriol, which binds to the vitamin D receptor (VDR) [13]. Whether produced by the skin through UVB light or from food, it was primarily known to support bone health by regulating calcium and phosphate metabolism, as shown in Fig. 2. Now, we know its non-skeletal functions affect the brain’s and muscles’ functioning [14]. There are many more functions of vitamin D than previously assumed. The VDR expressed in numerous cell types activates multiple essential genes of the immune system, making it a molecule of intensive study [15]. Additionally, vitamin D alleviates the progress of the proinflammatory T-helper 17 (Th17) and T-helper 9 (Th9) cells, regulates the growth of regulatory T cells (Tregs) cells, and promotes antibody production, particularly Immunoglobulin G (IgG) [16].

Furthermore, one of the most prominent nonclassical vitamin D actions has been explored for its modulation of the immune system via paracrine, intracrine, and endocrine mechanisms. Therefore, an inclusive understanding of vitamin D is needed. Other than vitamin D, metal ions also contribute to diverse functions related to immunity and are collectively studied under “metalloimmunology” [17]. Zinc is a significant micronutrient, whose homeostasis is regulated by antagonistic zinc transporters (ZnT) and ZIP proteins. Zinc is a part of humoral and cell-mediated immune signaling pathways, encompassing innate and adaptive immunity. Additionally, it has been reported that zinc modulates severe immune responses, by mitigating inflammatory reactions and has proven beneficial in several pathogenic respiratory disorders [18]. Remarkably, both vitamin D and zinc are known to safeguard immune tolerance and prevent overt reactions leading to severe pathology in several infectious diseases owing to their anti-inflammatory roles. Therefore, in this comprehensive review, we intend to provide an update on the essential findings and milestones in the discovery of vitamin D and zinc as immunomodulators with particular reference to respiratory infections and extrapolate their possible use in COVID-19 treatment to strengthen the immune system.

## Paths of Immunity Regulation: Vitamin D Receptors and Vitamin D Binding Proteins

**Fig. 3:**
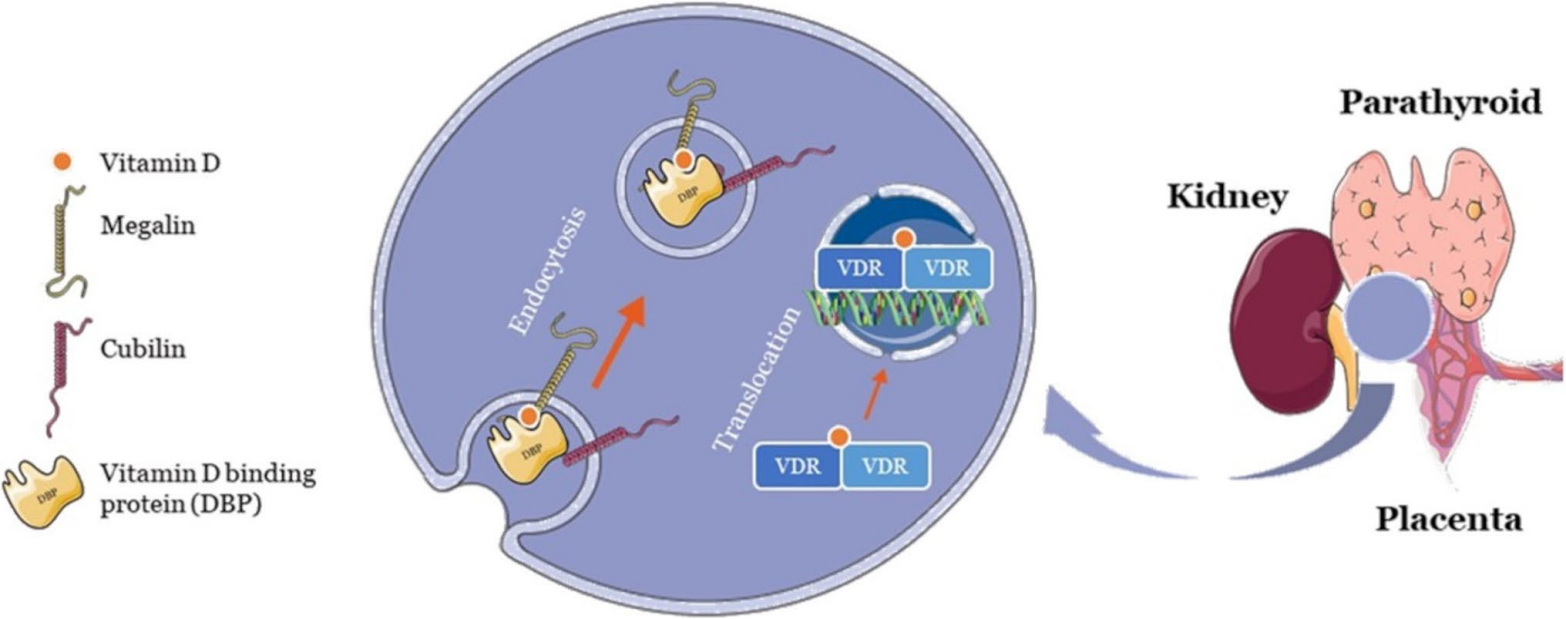
Transportation and mechanism of action of vitamin D on the placenta, kidney, and parathyroid cells

Vitamin D and its metabolites exist in bound form, though only free vitamin D can enter the cell and bind to vitamin D receptors. Approximately 85% is conjugated to the vitamin D binding protein (DBP) and 15% to albumin, while only 0.4% of total 1,25 (OH)_2_D_3_ and 0.03% of 25(OH)D_3_ are free. Endocytic receptors, megalin, and cubilin on the kidney, parathyroid, and placenta facilitate the entry of protein-bound vitamin D into the cells, where the DBPs act as ligands for these receptors (Fig. 3). Nevertheless, it is the free vitamin D-VDR complex that affects gene transcription. Hence, even the bound vitamin D is processed and freed [19]. Initially, as VDRs were found in nephrocytes and hepatocytes, it was believed that cholecalciferol underwent modifications in kidney and liver cells to become biologically active 1,25(OH)_2_D. However, now, vitamin D is reported for its various nonconventional functions. The first strong indication of the involvement of vitamin D in modulating the immune system comes from the discovery of VDRs in innate and acquired immune cell types, such as dendritic cells (DCs), T lymphocytes, B lymphocytes, and natural killer (NK) cells. Notably, these VDRs in numerous cell types, including immune cells, occur for some reason. Moreover, there are vitamin D–responsive genes distributed throughout the immune system, most of which can convert 25(OH)D to 1,25(OH)_2_D, reinforcing that vitamin D is required locally and thus produced locally for various functionalities [20]. Indeed, vitamin D is a pleiotropic molecule regulating immunity via diverse pathways and effector components.

### Vitamin D: the Quintessential Regulator of the Innate Immune System

**Fig. 4:**
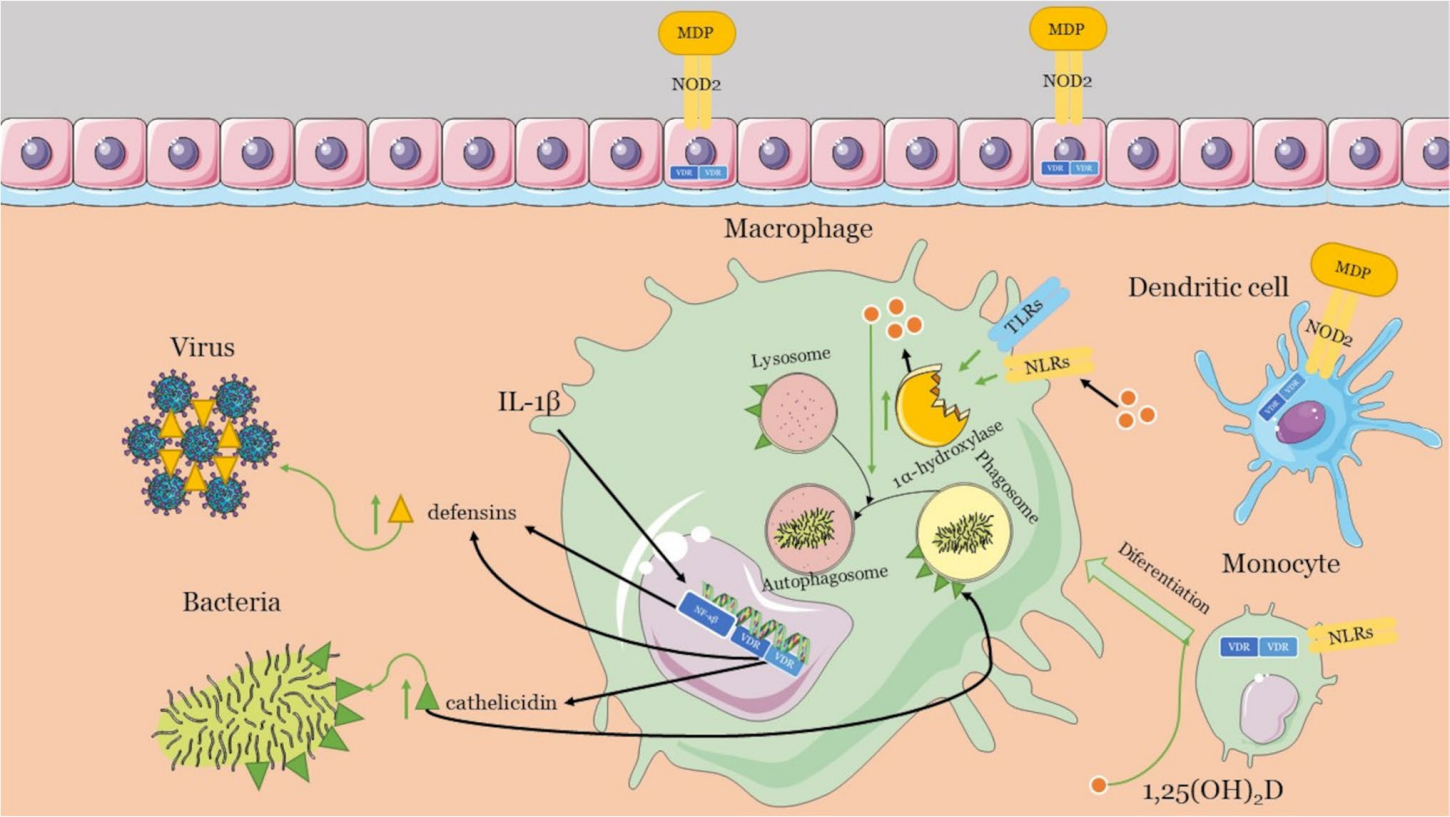
Role of vitamin D on the innate immune system

This nonspecific first line of the immune response against infection has several active functional components, such as DCs, macrophages, monocytes, granulocytes (neutrophils and eosinophils), and epithelial cells. Four classes of pattern recognition receptors (PRRs) help the cellular effectors of innate immunity to recognize pathogen-associated molecular patterns (PAMPs) and danger-associated molecular patterns (DAMPs). These are the Toll-like receptors (TLRs), the RLRs (retinoic acid-inducible gene 1 (RIG-1)-like receptors), the NLRs (nucleotide-binding oligomerization domain (NOD)-Leucine-rich repeats (LRR)–containing receptors), and the C-type lectin receptors (CLRs) [21]. Remarkably, vitamin D has been found to be crucial for the working of the innate immune system. As early as 1986, vitamin D receptors were found on monocytes. Later, it was established that monocyte differentiation into macrophages required 1,25(OH)_2_D. This active form, 1,25(OH)_2_D, is even synthesized inside macrophages by the action of its 1α-hydroxylase isoenzyme [22]. Pointing to the importance of vitamin D, reports have established that macrophages activated through TLR and NLR engagement show increased 1α-hydroxylase isoenzyme activity (Fig. 4). Engaged PRRs serve the innate immune system to curb infection by promoting proinflammatory responses and chemicals as barriers that include microbicidal peptides such as cathelicidins and defensins [23]. Nevertheless, they also assist in stimulating the adaptive immune system cells to eliminate pathogens. The activity of nucleotide-binding oligomerization domain-containing protein 2 (NOD2), one of the NLRs, is also enhanced by 1,25(OH)_2_D. Notably, the NOD2 ligand is muramyl dipeptide (MDP), a bacterial cell wall component, indicating the various pathways of vitamin D action in the innate immune responses to bacteria. In addition, the antimicrobial activity of 1,25(OH)_2_D is also attributed to the production of reactive oxygen species. In addition to monocytes and macrophages, DCs and epithelial cells express NOD2 and VDR.

Antimicrobial peptides (AMPs), expressed in immune cells and surfaces, form the first line of defense. Cathelicidin belongs to the microbicidal peptide family and is represented by the lone member hCAP-18 (human cationic antimicrobial protein) in human beings. This precursor protein is activated by cleavage to form LL-37, which is antimicrobial against many viruses, bacteria, and fungi [24]. The 25-hydroxyvitamin D [25(OH)D] level in the plasma and cathelicidin positively correlate, as the amount of cathelicidin produced by monocytes decreases with a drop in the vitamin D levels [25, 26]. Mechanistically, the vitamin D response element (VDRE) located upstream in the promoter region of cathelicidin, when bound by VDR, increases the expression of the messenger RNA (mRNA) and proteins of these peptides [27].

Furthermore, it helps in phagocytosis by enabling the fusion of the inactive phagosomes with active autophagosomes [28]. Cathelicidins help fuse an autophagosome to a lysosome, promoting autophagy through the formation of autolysosomes [29]. Thus, 1,25(OH)_2_D promotes cathelicidin and defensins production to mount an immune reaction against infection, regulating immunity. Unlike lone cathelicidin, two groups of defensins (alpha- and beta-defensins) are known to regulate immunity in humans. Mechanistically, they lead to viral aggregation and inhibit viral infection by preventing replication. β-defensin 2 is one such beta-defensin with a VDRE sequence in its promoter. It is stimulated indirectly by 1,25(OH)_2_D helped by IL-1β, which activates the NF-κB response elements near VDRE [30]. As both cathelicidins and defensins are regulated by vitamin D, it is an essential innate mechanism to fight infections. Autophagy plays a vital role in the functioning of the innate immune system against infections. Recently, it has been established that many of the signaling pathways in autophagy are modulated by calcitriol [31]. Important autophagy molecules involved in immunity-related autophagic processes are B cell lymphoma 2 (Bcl-2), beclin-1, the class III phosphatidylinositol 3-kinase complex (PI3KC3), mammalian target of rapamycin (mTOR), cathelicidin, calcium, and cyclin-dependent kinase. Autophagy starts with nucleation, and 1,25(OH)_2_D promotes nucleation by elevating the Beclin-1 PI3KC3 protein. Moreover, the inhibition of Bcl-2 through VDR with an increase in cathelicidin suppresses nuclear factor kappa B (NF-kB) signaling and promotes nucleation. Furthermore, 1,25(OH)_2_D increases autophagy by inhibiting the mammalian target of rapamycin (mTOR) via a decrease in cytosolic calcium. An increase in NOD2 levels induced by 1,25(OH)_2_D leads to the recruitment of autophagy-related genes (ATG16). ATG16 is one of the core autophagy proteins, helping in the elongation of autophagic vacuoles. In addition, even vacuole maturation, its lysosomal fusion, and degradation are also aided by 1,25(OH)_2_D. Interestingly, vitamin D3 acts as a dual regulator, wherein it is also proposed to decrease autophagy under certain conditions via TNF-α, NF-kB, or interferon-gamma (IFN-γ). Reports showed that calcitriol inhibited autophagy and protected cells against autophagic cell death via a cyclin-dependent kinase, p19INK4D [32, 33].

Thus, vitamin D3–mediated autophagy attempts to causes the death of microorganisms such as bacteria, viruses, and fungi, where cathelicidins, defensins, and reactive oxygen species (ROS) are regulated by calcitriol. This response is not limited to monocytes. Calcitriol also regulates other cells, such as natural killer cells, keratinocytes, epithelial cells, and decidual cells to influence the innate response, which is crucial in mitigating early infections [34]. Collectively, the impaired acquired immune responses and uncontrolled inflammatory innate responses to SARS-CoV-2 may cause cytokine storms [35]. Notably, vitamin D can shift the immune response to the anti-inflammatory side by suppressing the synthesis of proinflammatory Th1 cytokines, such as TNF-α and IFNγ, and enhance the manifestation of anti-inflammatory cytokines at the same time [36, 37]. There is yet another microRNA (miRs)-based regulation by which vitamin D regulates the innate response to viral pathogens. Mechanistically, miRs in contact with RNA-induced silencing complex (RISC) aid in gene silencing and expression by binding to the complementary sequences on the target RNA and vitamin D [38]. On the other hand, almost all the innate immune mechanisms discussed above converge on the formation of inflammasomes such as the NOD-like receptor family, pyrin domain containing 3 (NLRP3) inflammasome, which are large multiprotein oligomer assemblies initiated by the pattern recognition receptors. The priming and stimulation of NLRP3 is a tightly controlled process whose disturbance leads to several inflammatory disorders with a surge in proinflammatory cytokines, especially IL-1β and IL-18. Interestingly, vitamin D is a negative regulator of this inflammasome activity. Specifically, VDR interacts with NLRP3, preventing its deubiquitination by an enzyme BRCC3 and hampering its activation [39]. As NLRP3 participates in pyroptosis, which is required to clear infections, vitamin D is essential even for fine tuning pyroptosis, so it does not harm cellular physiologies.

### Dendritic Cells: at the Crossroads of Innate and Adaptive Immunity and the Effect of Vitamin D

Both the innate and adaptive components are imperative for proper immune system functioning. Although DCs are the portion of the innate immunity acting as the watch guards, they also serve as the channel between the two arms of the immune system. Processing and presenting antigens on the surface of DCs aided by their dendrite-like cytoplasmic processes initiate and amplify the adaptive response by triggering the T lymphocytes. DCs are cells that squeeze their dendritic processes through the tight junctions to catch, process, and express the antigens to instigate naive T cells [40]. In response to bacterial and viral infections, the DCs squeeze into the draining lymph nodes and undergo maturation from immature DCs (iDCs) to antigen-presenting cells (APC) [41]. Meanwhile, they produce proinflammatory cytokines TNF-α and IL-12, chemokines, and other co-stimulatory molecules. The DC pool is constantly replenished from bone marrow and even monocytes. In particular, active vitamin D 1,25(OH)_2_D_3_ and its receptor have a profound modulatory effect on DC maturation, wherein they mitigate antigen presentation and chemokine and cytokine production. The vitamin D–VDR complex has a varied impact on several groups of pathways, such as NF-kB and glucocorticoid receptor (GCR) signaling [42]. It suppresses the activation of antigen-specific CD8 T cells and helps to produce T reg cells. In total, vitamin D induces the anti-inflammatory and tolerogenic components of DCs, evident from the reduced cluster of differentiation 40 (CD40), cluster of differentiation 80 (CD80), and cluster of differentiation 86 (CD86), and IL-12, with boosted IL-10 levels, which prevents a flare-up adaptive immune response. The 1,25(OH)_2_D_3_-VDR complex may have differential effects on varied groups of cells, including DC interactions with transcription factors such as NF-κB or NFAT, leading to anti-inflammatory effects [43].

### Vitamin D is the Regulator of the Adaptive Immune System

The innate immune system fortifies the actions of the adaptive immune system to mount a full-fledged immune response against foreign invasion. This system primarily serves two purposes: first, containing the infection and, secondly, evolving immunological memory for future responses. Specialized leukocytes, such as the B and T lymphocytes, are the effectors in the humoral and cell-mediated adaptive immune response, respectively. However, the major histocompatibility complex (class I MHC and class II MHC) molecules present antigens to specific T cell types for further action. Subsequently, an activated T cell synthesizes the enzyme CYP27B1 to convert inactive calcidiol also known as 25(OH) Cholicalciferol into active calcitriol. Moreover, it even expresses vitamin D receptors to allow the binding of 1,25(OH)_2_D, critical for T cell functioning. In vitro, calcitriol has been reported for its noticeable inhibitory effect on adaptive immunity [44], since it attaches to the VDRE elements in the promoters of several genes, including the proinflammatory cytokines such as IL-2 and IFNγ, by complexing with VDR. Then, it blocks T cell proliferation and cytotoxic responses, along with the expression of proinflammatory cytokines to tilt the immune response to the Th2 type; vitamin D not only affects the transcription of genes through VDRE but also alters the cytoskeleton rearrangement, necessary for the integrin-mediated adhesion of CD4(+) T cells [45]. Furthermore, this prevents the migration of T cells to lymph nodes via chemokine inhibition. Thus, along with antiproliferative effects, the active vitamin D also regulates T cell migration and development [34, 45]. Notably, as memory T cells have a higher expression of VDRs, the antiproliferative effects are higher in memory T cells. Collectively, calcitriol is immunosuppressive in action for T cells. Moreover, VDR and vitamin D signaling regulate the proliferation of B cells. Vitamin D also caps the B cell and plasma cell differentiation regulating immunoglobulin secretion (IgG and IgM). As with memory T cells, B cell differentiation and apoptosis are partially controlled by vitamin D signaling [46].

### The Varied Roles of Zinc in the Innate and Adaptive Immune System

**Fig. 5:**
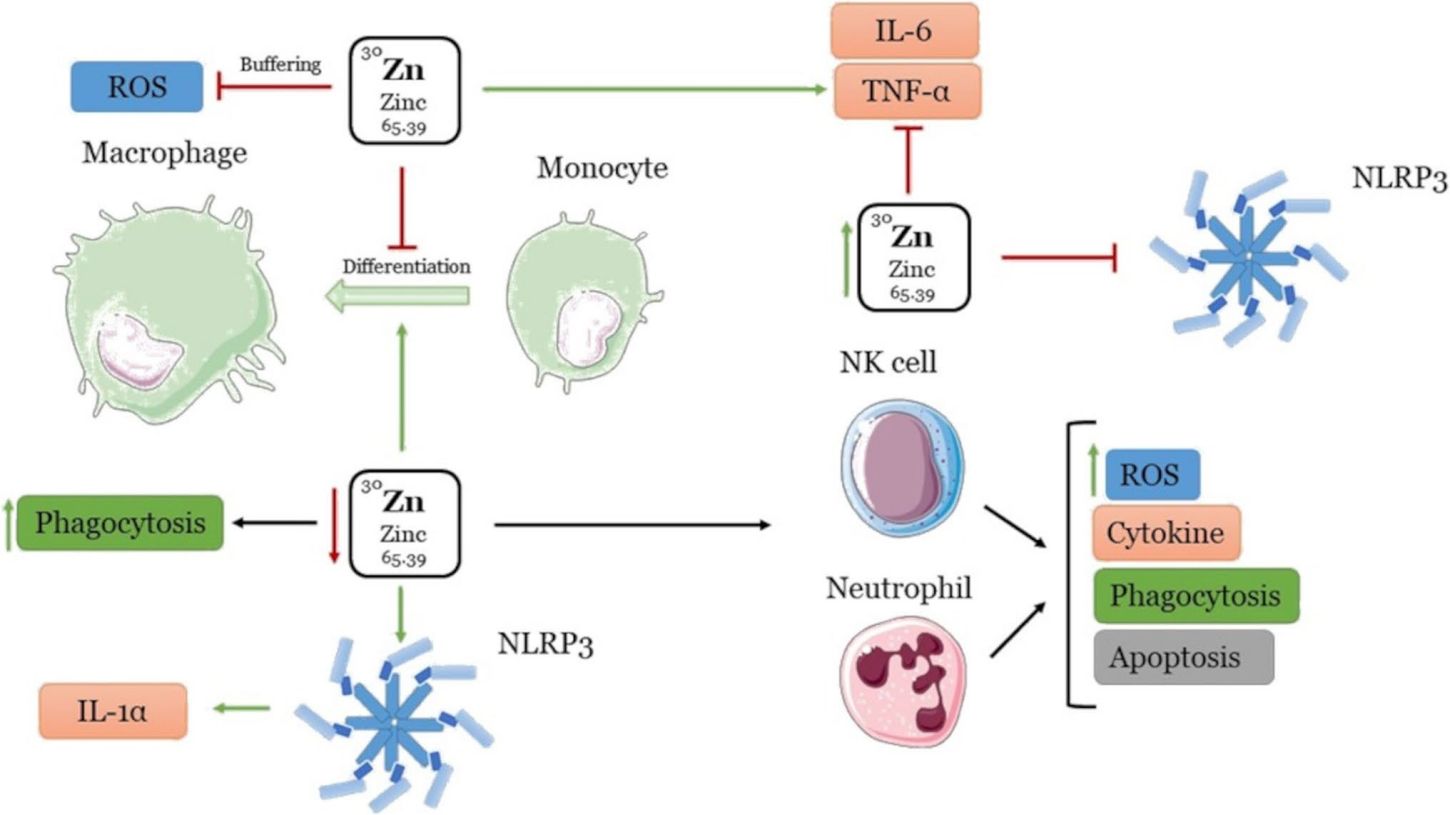
Effects of zinc levels on innate and adaptative immune response

Zinc is a crucial factor for various cellular signaling cascades in humans. The unearthing of ZIP and zinc transporters (1-14) revealed that they help to regulate the influx and efflux of zinc from the cytoplasm, respectively, which led to the notion of zinc homeostasis in inflammatory responses. Furthermore, mounting pieces of evidence reinforce the role of zinc homeostasis in modulating immunity. When it disappears, it impacts several infections and diseases such as diabetes, atherosclerosis, rheumatoid arthritis, impaired cognition, and macular degeneration [47]. More importantly, there is a complex interplay of diverse mechanisms, wherein zinc exhibits anti-inflammatory, proinflammatory, and regulatory roles within the immune system. Zinc’s changing levels affect innate and adaptive immunity, as it forms an essential constituent of immune cells. Remarkably, zinc inhibits the differentiation of innate immune cells such as monocytes and impairs their functions. Thus, its insufficiency promotes the formation of macrophages, indicating that the absence of zinc enhances the innate immune abilities of monocytes while restricting the adaptive responses [48]. Additionally, in a zinc-deficient scenario, pathogens are cleared off by increased oxidative stress and phagocytosis rather than by inflammatory cytokines [49]. Furthermore, zinc is known to aid in lipopolysaccharides (LPS)-induced toll-like receptors (TLR) signaling to promote proinflammatory cytokines. However, an optimum zinc level is needed to mount an effective immune response (cytokines such as IL-6 and TNF-α) against invading pathogens. Conversely, high concentrations of intracellular free zinc prevent the same [50], and it also regulates the activity of NK cells and neutrophils along similar lines. Moreover, low levels of zinc are associated with increased ROS, dysregulated cytokine production, impaired phagocytosis, and apoptosis. When NF-kB signaling induces inflammatory responses during infection or oxidative stress, zinc helps to combat the harmful effect of free radicals by exhibiting an “oxidation buffering” property [51]. In addition, zinc deficiency has also been reported to lead to NLRP3 inflammation activation resulting in an upsurge in the proinflammatory IL-1α cytokine (Fig. 5). Zinc supplementation exerts its anti-inflammatory response by dampening NLRP3 [52].

### The Role of Vitamin D and Zinc in Modulating the Immune Response in Respiratory Diseases

Vitamin D is actively involved in all the stages of the host defense mechanisms. Maintaining the barrier between epithelial cells, by protecting tight junctions, adherent junctions, and gap junctions, modulates the innate and adaptive reaction to pathogens [53]. Around two hundred vitamin D–responsive genes have been reported in monocytes and macrophages. In contrast, leukocytes have around 60 vitamin D–responsive genes, suggesting strongly that vitamin D has significant properties on both the maintenance and repair of the immune system in normal or diseased conditions, when the immune responses disappear [54]. As early as 1840, it was shown that cod liver oil was beneficial against tuberculosis. It took nearly 100 years for the effect to be ascribed to vitamin D. An exploratory journey followed to decipher the role of vitamin D in bacterial and viral respiratory infections and the immunity face-off [55]. Several bacterial and viral respiratory tract infections, such as asthma, tuberculosis, chronic obstructive pulmonary disease (COPD), and wheezing are linked to vitamin D deficiency [56, 57]. Apart from these, viral infections such as hepatitis, herpes, dengue, and influenza may have greater incidence and severity in populations with inadequate 25(OH)D levels [58]. Notably, in animal models of microbial acute lung injury (ALI) and acute respiratory distress syndrome (ARDS), the renin–angiotensin system (RAS) was central to the pathogenicity, as it affected the vascular tone and permeability in addition to the fibroblast activity and alveolar cell survival. Moreover, as the RAS played a vital role in maintaining circulatory homeostasis, and vitamin D modulated the expression of the RAS, together with angiotensin converting enzyme 1 (ACE1) and angiotensin converting enzyme 2 (ACE2), vitamin D became even more critical [59]. Vitamin D is converted to its active form by the respiratory epithelium locally as a host defense mechanism, wherein it increases the expression of ACE2. Then, the ACE2 enzyme, which acts as a negative regulator of RAS, prevents lung inflammation by regulating the permeability, edema, and lung tissue oxygenation [60]. Thus, an adequate supplementation of vitamin D proves beneficial in treating respiratory infections. Here, as disruption of the RAS functioning leads to acute inflammation and cytokine production, vitamin D deficiency adds to the distress. An infiltration of immune cells characterizes respiratory tract infections in respiratory tissues and proinflammatory responses, including cytokines (IFN-1, TNF-alfa, and IL-6) and chemokines (CXCL8 and CXCL10). Mechanistically, besides preventing immune cell infiltration and modulating the cytokine response, vitamin D also kills the pathogens through diverse pathways [61]. Reportedly, upper respiratory tract infections (URTIs) decreased with increased serum vitamin D (25-OHD) levels. Still, it was also reported in specific populations that 100,000-IU/month of vitamin D3 did not reduce the frequency or severity of URTIs [58]. Nonetheless, several meta-analyses and systematic reviews have also established that lower 25(OH)D levels were linked with an increased risk for respiratory syncytial virus-lower respiratory tract infections (RSV-LRTI) and tuberculosis in infants [57, 62]. Moreover, its supplementation ameliorated the symptoms in patients with a lower than usual (25 nmol/L) vitamin level [63]. Furthermore, reports showed that vitamin D supplementation in children could be a robust means of preventing the incidence of respiratory infections. In school children, a randomized, double-blind, and placebo-controlled trial showed that vitamin D supplementation (1200 IU/d) in winter prevented influenza A infection in children [64].

Furthermore, owing to its role in innate and adaptive responses, zinc insufficiency has been linked with the severity of numerous respiratory tract infections, including tuberculosis, pneumonia, influenza, etc. [65–67]. As with vitamin D, zinc regulates inflammation and prevents recurring, acute, and chronic respiratory tract infections, providing beneficial disease outcomes on supplementation. Moreover, zinc helps maintain the respiratory epithelium’s homeostasis by regulating the apoptosis of epithelial cells through pro and active caspase-3 levels, thus fortifying the innate defense mechanisms [68, 69].

### The Standing of Vitamin D and Zinc as a Preventive and Therapeutic Answer to COVID-19

Vitamin D can be an old answer to a new question posed by the COVID-19 pandemic. Around a century ago, when the world was hit hard by influenza, which took millions of lives with the first and second waves, the reasons were attributed to adverse immunological responses such as cytokine storms. The importance of vitamin D in boosting the immune system and preventing the risk of many respiratory diseases has been widely studied since 2001. The outbreak of COVID-19 revealed the antioxidant and anti-inflammatory properties of vitamin D. In vitro experiments elaborated the antiviral role of 1,25(OH)_2_D_3_, as it attenuated lipopolysaccharide-induced lung injury by regulating the ACE2 through the renin–angiotensin system (RAS). This mechanism of the RAS system is of prime importance in the disease severity of COVID-19, at low serum levels of vitamin D [70].

Researchers worldwide have established a link between low 25(OH)D levels and the disease’s increased severity and the fatality of respiratory diseases such as influenza, which appears to be repeating itself now with the SARS-CoV-2 infection [71]. In a recent retrospective cohort study, Meltzer et al. found that out of 489 patients, whose 25(OH)D levels were tested before COVID-19, the patients with a vitamin deficiency were at almost a double risk of contracting the disease [72]. In total, it was established that inadequate 25(OH)D levels led to lethal immune system dysregulation, including innate and adaptive responses resulting in lung damage due to cytokine storms, bad cell repair, and apoptosis of epithelial cells, which can have severe implications for COVID-19 [73, 74]. The role of low vitamin D levels in lung inflammation was identified through in vivo clinical trials on mouse models subjected to the influenza A virus subtype (H1N1), H1N1 influenza, and SARS-CoV-2 infection. The vitamin D levels were determined, and the mice were grouped into vitamin D–sufficient and deficient mice. These two groups were then exposed to the H1N1 viral infection; the vitamin D–sufficient mice group survived the viral attack. Thus, this study elaborated that a deficiency of vitamin D can increase the disease severity, while the sufficiency or supplementation of vitamin D reduces the inflammation caused by pandemic viral infections [75].

Moreover, evidence suggests that the incidence, prevalence, and relentlessness of the COVID-19 pandemic correlate with vitamin D deficiency in specific high-risk category patients with obesity, diabetes, and hypertension. COVID-19 is linked to vitamin D deficiency through ethnicity, geography, and seasonal variation [76]. Notably, researchers worldwide have reported that the Black, Asian, and minority ethnic (BAME) population, especially men, have been more severely affected by COVID-19 [77]. Moreover, vitamin D’s sex-related differences in immune modulation are attributed to the female hormone estradiol. The anti-inflammatory response evidence mounted in the presence of vitamin D is strengthened by the administration of estradiol and is more significant in women than men. Specifically, the production of IFN-γ and IL17 is inhibited [78]. Transcriptome analysis showed that the number of genes prominently affected by vitamin D was around four times greater in women than men.

Moreover, these genes affected by vitamin D have been linked with cytokine- and B cell–mediated immune signaling. Takahashi et al. [79] reported that during SARS-CoV-2 infection, the immunological response differed with sex differences for SARS-CoV-2 infection. Vitamin D and estradiol act synergistically to mount an anti-inflammatory response. Estradiol increases the vitamin D function and the expression of the vitamin D receptor, resulting in a more powerful anti-inflammatory response in females than males, silencing the cytokine storm. Vitamin D also regulates the estradiol level. Thus, in fertile women, the crosstalk between vitamin D and estradiol plays a protective role. This partly explains the difference in COVID-19 incidence and severity with age and sex. However, any therapeutic approach should consider that women have more robust immune reactions and are more prone to autoimmunity than men. In this regard, due to its differential regulatory roles in different sexes, vitamin D holds promise to tackle the seriousness of the disease [79].

Due to various reasons, such as less physical activity and sun exposure coupled with the inefficiency of the skin in synthesizing vitamin D from the radiation, vitamin D levels decrease in older people compared to youth [80]. As immunity declines with age, more COVID-19–related deaths in elderly patients can be due to inflamm-aging, the unwarranted and skewed immune response due to aging. As the antigenic load increases with age, a concomitant decrease in the body’s overall capacity to neutralize the antigens leads to immunosenescence and increased vulnerability to various diseases and infections. Moreover, the cytokine storm responsible for COVID-19 severity in aged individuals is also favored by inflamm-aging [81]. Vitamin D regulates inflammation, and low vitamin D levels are linked with a hampered immune response. An optimum vitamin D level seems necessary to mitigate the adverse outcomes of inflamm-aging and COVID-19. Vitamin D inhibits the inflammatory response via IL6 and may mitigate COVID-19 symptoms [82]. Calcifediol, an activated form of vitamin D, abated the severity of the disease in a pilot randomized controlled trial [83]. In a study, the ability of vitamin D to diminish the inflammatory responses generated in COVID-19 patients was evaluated by the expression analysis of proinflammatory cytokines. It was revealed that vitamin D–treated patients possessed low levels of cytokines and COVID-19 inflammatory markers, suggesting it had immunomodulatory behavior [84].

An epidemiological study was conducted in India to find an association between hypo-vitaminosis and COVID-19. The study included 156 COVID-19–positive patients and 204 controls with no detection of the COVID-19 virus, and the level of serum vitamin D was determined through a chemiluminescence-based immunoassay analyzer. It was reported that the level of vitamin D was below 10 ng/mL in the COVID-19 patients tested as compared to the controls [85]. Another study evaluated the demographic data of all the patients that significantly showed a higher risk of COVID-19 disease due to their increased age, low vitamin D levels, smoking, and alcohol intake. This study showed the importance of vitamin D supplementation as a primary care strategy to control the health risks caused by COVID-19 [86]. However, the results of the small RCTs were inconsistent because of factors that need to be monitored carefully, such as the nutritional status of the subjects. Hence, there is still a need to carry out large trials with properly matched subjects and controls with appropriately measured parameters. Nevertheless, this does not deny the fact that vitamin D is looked upon as a promising treatment in pandemics such as influenza and COVID-19 [53, 87].

Previous studies have elaborated a significant role for hypovitaminosis D in COVID-19 disease infection. In a recent research study, the association of low vitamin D levels in COVID-19 patients was analyzed against a set of inflammatory markers produced as an immune response. This study on the correlation between low levels of 25 hydroxy vitamin D (25 OH vitamin D), and disease severity was conducted on 93 patients suffering from pneumonia as a result of COVID-19 infection. About 65% of the COVID-19 patients showed hypovitaminosis D (less than 20 ng/mL) and elevated levels of the C-reactive protein, interleukin-6, tumor necrosis factor-α, d-dimers, and interleukin-10 markers. An inverse correlation was observed between the inflammatory biomarkers and the levels of 25 OH vitamin D, showing the importance of vitamin D as a marker in disease management strategies [88, 89]. There has been a steady rise in research establishing vitamin D as an immunomodulator. Vitamin D decreases ACE and angiotensin II expression and increases ACE2, ameliorating inflammatory lung damage. Additionally, VDR knockouts showed aggravated lung injury symptoms, sepsis, and mortality [90, 91]. In a clinical research trial, the effect of vitamin D deficiency on the lung involvement, disease duration, and mortality rate was reported in 65 elderly (mean age 76 ± 13 years) COVID-19 patients, and it confirmed that 25OH-vitamin D serum deficiency was associated with more severe lung involvement, longer disease duration, and the risk of death in elderly COVID-19 patients [92]. Vitamin D is considered vital for the proper functioning of lungs, as it regulates the secretion of cathelicidin, DCs, chemokine production, and T cell activation. Infection with COVID-19 disturbs the serum vitamin D levels and halts the immunity and radiologic lung involvement, as analyzed by computed tomography experiments [92].

Furthermore, the cytokine storm witnessed in COVID-19 can be modulated by vitamin D by tweaking the reaction concerning an anti-inflammatory Th2-type immune response. Specifically, it prevents the synthesis of proinflammatory Th1 cytokines such as TNFα and IFNγ [45, 93]. The serum levels of vitamin D were lower in a small group of COVID-19–positive patients in the USA. Reports have suggested an inverse relation between the severity of PD and vitamin D levels [94]. There is a high prevalence of vitamin D deficiency in Parkinson’s disease (PD). Moreover, females are at a lower risk of PD than men because of the hormone estradiol [95]. Further, it has reported that there can be higher incidence of Parkinson’s because of COVID-19. Altogether, there seems to be an interplay between PD, vitamin D, and COVID-19. Supplementation with vitamin D to meet the deficiencies has been reported to reduce the disease severity in PD. A study of Parkinson’s disease patients in Italy stated a reduced incidence of COVID-19 in patients with vitamin D supplementation [72, 96]. Recently, Maghbooli et al. [97] discovered that as little as 30 ng/mL of 25-hydroxy vitamin D reduces the clinical severity of the COVID-19 disease. Furthermore, a cohort study by Kaufman et al. established that low circulating levels of 25-hydroxy vitamin D caused a high risk of COVID-19 [98].

A clinical trial to identify the role of vitamin D supplementation in COVID-19 disease prevention was conducted. COVID-19–negative health care workers were selected from different hospitals of Mexico, and a dose of 4000 IU vitamin D was supplemented for 30 days. RT-PCR analysis revealed a lower rate of COVID-19 infection in these highly exposed workers, confirming the positive feedback of vitamin D supplementation in COVID-19 disease prevention [99].

Another controlled study showed the same results when a high dose, i.e., 10,000 IU per day of cholecalciferol, was given to COVID-19 affected patients. It was revealed that a high dosage supplementation of vitamin D also caused an increased synthesis of anti-inflammatory cytokines, i.e., IL-10 and CD-4 cells, with enhanced antiviral cytotoxic activity in the body. Additionally, patients with acute respiratory distress syndrome and supplementation with 10,000 IU cholecalciferol were reported to recover quickly [100]. Our study has shown the importance of vitamin D supplementation as a primary care approach to control the health risks caused by COVID-19. Many clinical trials [101–103] with the supplementation of vitamin D in response to COVID-19 were performed, and none of them reported any adverse side effects as a result of this treatment. Zurita-Cruz et al. [104] reported that supplementation of vitamin D decreased the COVID-19 disease progression even in pediatric patients, with no serious health complications, increased oxygen requirement, or death. Thus, these studies suggest the usage of vitamin D supplementation even in high doses (doses of 1000 IU/day (children < 1 year) or 2,000 IU/day (from 1 to 17 years)) without side effects [104].

Furthermore, zinc homeostasis and vitamin D functioning are linked. Meanwhile, while zinc intensifies the activity of specific vitamin D3–dependent promoters, on the other hand, vitamin D augments the expression of zinc transporters such as ZnT10 and thus aids in maintaining zinc homeostasis. Moreover, as the vitamin D receptor (VDR) can bind zinc, its concentrations regulate the expression of vitamin D–dependent genes [105]. Remarkably, zinc contributes to the type 1 interferon response, the regulation of the IL-6 levels, and the prevention of neutrophil migration, known to worsen lung injury during SARS-CoV-2 infection [106–108]. Hence, zinc can be used as an adjuvant with vitamin D to orchestrate the immune responses during SARS-CoV-2 infection. These results come from the studies that attributed vitamin D and zinc deficiency to similar adverse outcomes in COVID-19 [109]. Considering the current devastation due to the pandemic, vitamin D and zinc supplementation can serve as an alternative treatment.

A research study was conducted in order to determine the serum vitamin D and zinc levels in children (1–18 years) diagnosed with COVID-19 viral infection. In total, 88 diseased and 88 healthy controls were selected, and significant results were obtained, i.e., there was a lowered concentration of serum vitamin D and zinc levels in the children with COVID-19 as compared to the control [110]. The literature survey analysis revealed the importance of micronutrients, i.e., vitamin D and zinc deficiency dysregulated the host response generated due to COVID-19 infection. Many randomized clinical trials [102, 103, 110] have been performed to determine the combined effect of vitamin D and zinc supplementation in disease recovery and mortality. Moreover, this combined supplementation was compared with the standardized protocols followed in hospitals, and significant positive recovery rates were observed in favor of zinc and vitamin D supplementation as an addition to the medical therapy [103]. Owing to their low toxicity, wide availability, and few side effects, the recent data on vitamin D and zinc’s role convey the need to revisit the possibilities of vitamin D and zinc supplementation in COVID-19 therapeutics with simultaneous intricately designed studies and clinical trials [111].

## Conclusion

As there are not many peer-reviewed published clinical studies, a need for research still exists to determine the exact methods and dosages of vitamin D and zinc supplementation useful for combating COVID-19’s severity and incidence. There is still a lack of complete understanding of the intricacies of how SARS-CoV-2 infection is modulated by both vitamin D and zinc status. As there is an extensive diversity in the reference levels of vitamin D with VDR polymorphisms in the population worldwide, the research needs to be standardized at all steps, from measuring the serum levels of vitamin D to the genetic variations. However, because newer vitamin D functions are gaining a foothold, and the virus SARS-CoV-2 is still being explored, it is imperative to understand the biological mechanisms in detail to draw discrete connections between COVID-19 and vitamin D, as well as for zinc. Whether vitamin D can be used as a drug still needs to be determined in larger trials. Vitamin D and zinc supplementation to address the deficiencies in different populations can help to combat the outcomes of pandemics such as COVID-19.
